# *Gossypium hirsutum* gene of unknown function Gohir.A03G0737001 encodes a potential Chaperone-like Protein of protochlorophyllide oxidoreductase (CPP1)

**DOI:** 10.17912/micropub.biology.000867

**Published:** 2023-07-30

**Authors:** Alana N. Osborne, Andrew Osagiede, Amanda R. Storm, Amanda M. Hulse-Kemp, Angela K. Stoeckman

**Affiliations:** 1 Chemistry, Bethel University, Saint Paul, Minnesota, United States; 2 Biology, Western Carolina University, Cullowhee, NC; 3 Genomics and Bioinformatics Research Unit, USDA-ARS, Raleigh, NC; 4 Department of Crop and Soil Sciences, North Carolina State University, Raleigh, North Carolina, United States; 5 Chemistry Department, Bethel University, Saint Paul, Minnesota, United States

## Abstract

A gene of unknown function identified in *Gossypium hirsutum* , Gohir.A03G0737001.1, was studied using sequence and bioinformatic tools. The encoded protein (referred to here as GhCPP1-A0A1U8HKT6) was predicted to function as a Chaperone-like protein of protochlorophyllide oxidoreductase (CPP1), which is involved with initiation of photochemical reactions of chlorophyll biosynthesis. Sequence analysis indicates it is embedded in the chloroplast envelope membrane through four transmembrane regions and contains a J-like domain that is structurally similar to the J domain of DnaJ/Hsp40 “holdase” chaperone proteins.

**Figure 1. Sequence and Structure Characterization of GhCPP1-A0A1U8HKT6:**
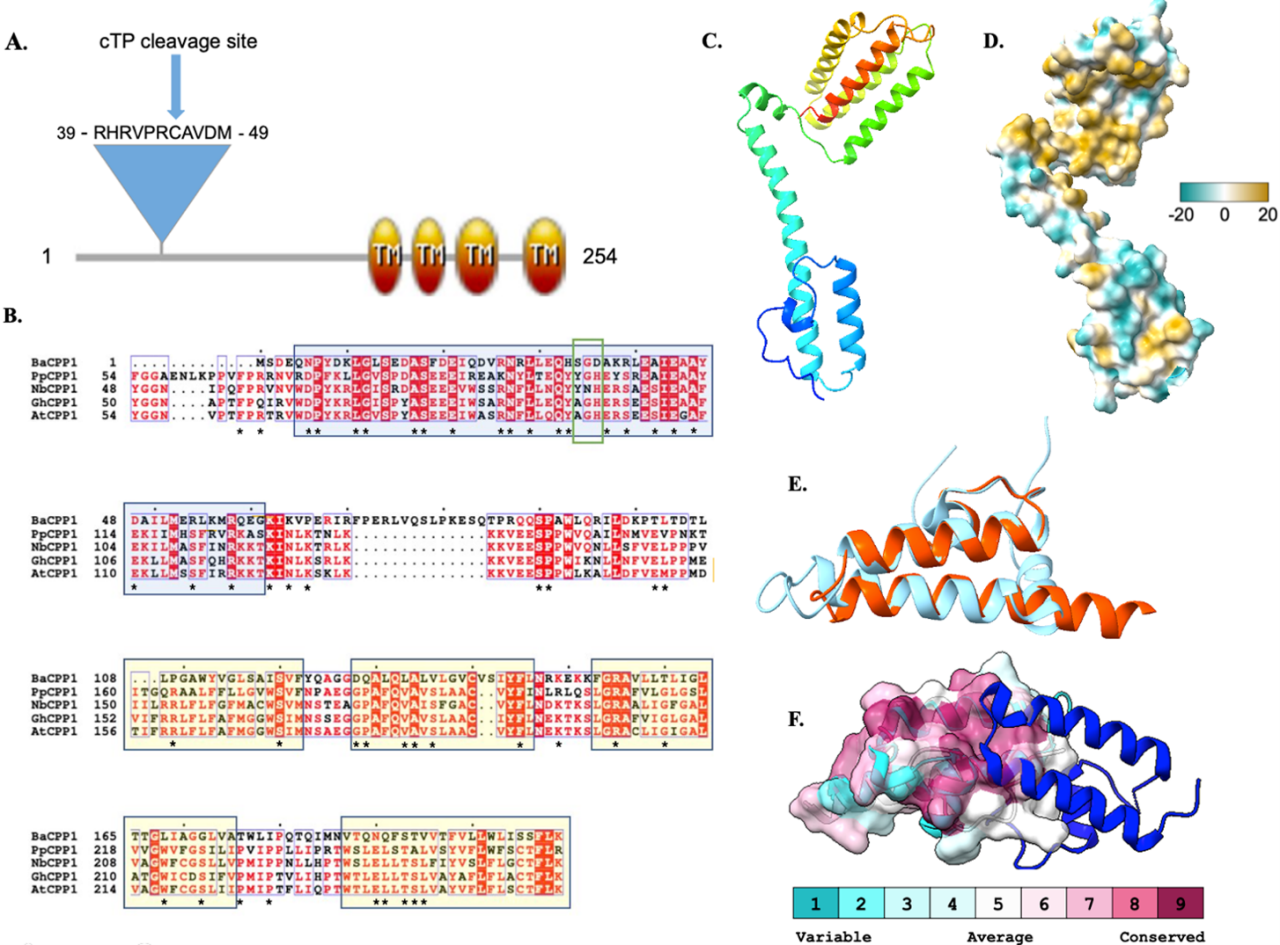
(A) Domain architecture of GhCPP1-A0A1U8HKT6 created using Prosite:MyDomains (Hulo et al. 2008) based on predictions from InterPro (Blum et al. 2021), TM indicates transmembrane, cTP is chloroplast transit peptide. (B) Multi-Sequence Alignment of homologs from *B. angustatum* (BaCPP1), *P. patens* (PpCPP1), *N. benthamiana* (NbCPP1), *A. thaliana* (AtCPP1), and *G. hirsutum* (GhCPP1) created with ClustalOmega (Madeira et al. 2019) and ESPript3 (Robert and Gouet 2014) highlighting locations of the AtCPP1 J-like domain (blue box) and GhCPP1 predicted transmembrane regions (yellow boxes). The green box indicates the corresponding location of the HPD motif present in DnaJ molecular chaperone J domains that is absent in J-like domains. ConSurf (Ashkenazy et al. 2016) identified highly-conserved residues are denoted with an asterisk. (C) AlphaFold model of GhCPP1-A0A1U8HKT6 amino acids 56-254 displayed by ChimeraX (Version 1.3, Pettersen et al. 2021; Jumper et al. 2021) in rainbow format with the N-terminus in blue and C-terminus in red. (D) Hydrophobic surface of the GhCPP1-A0A1U8HKT6 model structure with teal representing low hydrophobicity, and brown representing high hydrophobicity. (E) Proposed J-like domain of GhCPP1-A0A1U8HKT6 in orange (amino acids 63-119) overlaid with the J-domain of the human Tid1 protein (PDB ID: 2DN9) in blue. (F) Overlay of GhCPP1-A0A1U8HKT6 J-like domain (ConSurf coloring based on conservation) with (PDB 2GUZ) yeast mitochondrial import motor protein subunit TIM16 (light blue ribbon), showing potential interaction surface with TIM14 subunit (dark blue ribbon).

## Description

Introduction

Cotton plays a significant role in the United States economy as it is estimated to generate over 200,000 jobs and $25 billion dollars in annual revenue (Raper et al. 2019) . By the end of the 2022-2023 growing season, the United States Department of Agriculture (USDA) forecasts that the US will produce 18.2 million bales of cotton, a 4.5% increase of production from the 2021-2022 season (Johnson et al. 2022) . Research on cotton is essential as the price of cotton and the financial gain to the cotton grower is dependent on the fiber yield and quality. Recently, the genomes of five allotetraploid cotton species ( *Gossypium hirsutum* , *Gossypium barbadense* , *Gossypium mustelinum* , *Gossypium tomentosum* , *Gossypium darwinii* ) were sequenced (Chen et al. 2020) , and analysis of gene annotations revealed many genes of unknown function in common across the species.

We predict that one of these genes of unknown function, the *Gossypium hirsutum* gene Gohir.A03G073700 (CottonGen: mRNA ‘Gohir.A03G073700.1_UTX-TM1_v2.1" https://www.cottongen.org/bio_data/5920329 ), and associated protein (NCBI: XP_016666667.1; UniProt: A0A1U8HKT6) here referred to as GhCPP1-A0A1U8HKT6, is a chaperone-like protein of POR1 (CPP1). Angiosperms, such as *G. hirsutum* , require light for the biosynthesis of the green pigment chlorophyll. In one of the later steps of the biosynthetic pathway, protochlorophyllide is reduced to chlorophyllide with facilitation and catalysis by protochlorophyllide oxidoreductase (POR), a light-dependent enzyme (Lee et al. 2013) . Other oxygenic photosynthetic organisms also contain variations of POR genes, especially those with homology to angiosperms (Masuda and Takamiya 2004) . To complete chlorophyll biosynthesis, the POR enzyme must be post-translationally imported into the plastid (Reinbothe et al. 1995) . The chaperone-like protein of POR1 (CPP1), previously known as cell growth defect factor-1, (CDF1; At5g23040) has been shown to regulate POR permanence and function during this process (Lee et al. 2013) . Based on the function of CPP1 proteins, they are typically predicted to have subcellular localizations within chloroplast membranes with both cytoplasmic and transmembrane domains in their sequence. The GhCPP1-A0A1U8HKT6 protein evaluated here is likely a chaperone-like protein of POR1 found in *G. hirsutum* due to its structural and sequence features depicting similarity to already identified CPP1 proteins in angiosperms and related species.

Sequence Features

The InterPro webserver (Blum et al. 2021) identified the 254 amino acid GhCPP1-A0A1U8HKT6 protein as a member of the chaperone-like POR1 protein family (CPP1) (IPRO21788, PF11833). Sequence analysis of GhCPP1-A0A1U8HKT6 by the subcellular localization programs Plant-mPLoc (Chou and Shen 2010) and DeepLoc (Thumuluri et al. 2022) predicted the location of this protein to be the plastid. Furthermore, TargetP indicated that GhCPP1-A0A1U8HKT6 contains a chloroplast transit peptide cleavage site at amino acid position 45 (Almagro Armenteros et al. 2019) . This is in agreement with experimental localization of the Arabidopsis CPP1 protein (At5g23040) to the thylakoid and envelope membranes (Lee et al. 2013) . Transmembrane (TM) region predictions were performed using Phobius (Käll et al. 2007), TMHMM (Krogh et al. 2001) , and InterPro (Blum et al. 2021) . All three bioinformatic programs predicted that four TM regions were found in the GhCPP1-A0A1U8HKT6 protein, between amino acids 152-169, 175-191, 198-220, and 232-253. A domain architecture was created to visualize these sequence features ( **Figure 1A** ). Interestingly, *Arabidopsis thaliana* and *Nicotiana benthamiana* CPP1 proteins are indicated to contain three, not four, TM regions (Lee et al. 2013) . However, a homolog of *Arabidopsis* CPP1, At2g20920, has been identified to also contain four TM regions (Kawai-Yamada et al. 2014) suggesting a potential structural or functional difference between these family members.

Homology

Homology of GhCPP1-A0A1U8HKT6 to *Arabidopsis* proteins was assessed using the TAIR database (Berardini et al. 2015) , and the CPP1 protein (At5g23040, Q9FN50) was identified to be 80.3% identical with an 84% query cover. Additionally, the TAIR database indicates At5g23040 has orthologs in eudicots, monocots, lycophytes, bryophytes, and other photosynthetic organisms such as the single-celled green algae *Ostreococcus tauri* and *Chlamydomonas reinhardtii* . AtCPP1 promoter activity is low in the globular stage of *Arabidopsis* embryogenesis but increases throughout the heart and linear stages and persists through the mature embryo stage (Kawai-Yamada et al. 2014) . While homozygous knockouts of AtCPP1 are lethal (Lee et al. 2013) , suppression of AtCPP1 by RNA interference completely blocked the *in vitro* import of *Arabidopsis* PORA and PORB isoforms into isolated etioplasts (Reinbothe et al. 2015) . Although protein-protein interactions have been demonstrated between CPP1 and POR by fluorescent co-localization and immunoprecipitation (Lee et al. 2013) , CPP1 is not involved in the process of protochlorophyllide or NADPH binding to POR (Reinbothe et al. 2015) . Instead, due to the structural similarity between CPP1 and the DnaJ/Hsp40 protein family, it has been suggested that CPP1 acts as a “holdase” chaperone to permit preliminary cytoplasmic steps before initiating POR import into the plastid.

The “holdase” activity of the molecular co-chaperone DnaJ has been attributed to a J domain which is an approximately 70 amino-acid sequence containing four helices with a loop region between the second and third helix (Qiu et al. 2006) . The J domain is found across all kingdoms and plays an important role in organizing interactions with Hsp70 chaperone partners (Kelley 1998) . The presence of a functionally conserved His-Pro-Asp (HPD) motif within J domains of molecular chaperones, such as the human Tid1 protein, facilitates the ATPase activity of DnaK-like co-chaperones with which they interact (Lu et al. 2006) . The J-like domain is also found across kingdoms and, although very similar is structure to the J domain, the HPD motif is absent in J-like domain proteins such as CPP1, indicating these domains may perform a similar function but have diverged interaction partners. Amino acid sequences of CPP1 homologs from the eudicots *N. benthamiana* (NbCPP1; AEP68099) and *A. thaliana* (AtCPP1; At5g23040), the bryophyte *Physcomitrella patens* (PpCPP1; XP_024395830.1), and a cyanobacterium *Brasilonema angustatum* (BaCPP1; MBW4597090.1) were aligned with GhCPP1-A0A1U8HKT6 ( **Figure 1B ).** The blue box indicates the location of the J-like domain in *Arabidopsis, N. benthamiana,* and *P. patens* (Lee et al. 2013) and the green box indicates where the HPD motif would be located in a J domain (Qiu et al. 2006) although this motif is lacking in the J-like domain containing CPP1 homologs as well as GhCPP1-A0A1U8HKT6 (Lee et al. 2013) . Significant sequence conservation is observed from photosynthetic bacteria to eudicots in both the J-like domain and transmembrane domains (yellow boxes indicate *G. hirsutum* TM domains), with highly-conserved residues in GhCPP1-A0A1U8HKT6 identified by ConSurf (Ashkenazy et al. 2016) indicated with asterisks. The full ConSurf results are available as Extended Data.

Structural Features

AlphaFold and ChimeraX (Version 1.3, Pettersen et al. 2021; Jumper et al. 2021) were used to predict and visualize the model structure of GhCPP1-A0A1U8HKT6. **Figure 1C** depicts two high-confidence modeled regions between amino acids 56-119 and 150-254 and a low-confidence modeled region between them from amino acids 119-150. The structure is oriented vertically to show the cytoplasmic domain at the lower part of the image and the four transmembrane helices at the upper part of the image. The transmembrane domains exhibit expected high hydrophobicity as visualized by the ChimeraX surface calculation tool ( **Figure 1D,** same orientation as **1C** ). A structural overlay of the J-like domain (amino acids 63-119) of GhCPP1-A0A1U8HKT6 with the J domain of the human DnaJ family member Tid1 (PDB ID: 2DN9) displays a similar anti-parallel hairpin between the horizontally-displayed alpha helices II and III, although GhCPP1-A0A1U8HKT6 lacks the fourth helix of human Tid1 and has a shorter loop ( **Figure 1E )** . The structural similarity with key sequence differences between a cotton protein and human protein demonstrates this is a highly conserved structural domain that likely has developed a unique function.

When the DALI database (Holm 2020) was searched for proteins having a similar tertiary structure to the proposed J-like domain of GhCPP1-A0A1U8HKT6, the second highest similarity hit (Z-score of 7.9 and RMSD score of 1.5) was with the J-like domain of yeast TIM16 that is part of the translocase of the inner membrane protein complex, TIM16/TIM14 (PDB ID: 2GUZ). The TIM16/TIM14 heterodimer complex serves as a co-chaperone duo, a critical part of the translocase import motor responsible for piloting translocation proteins into the mitochondria (Mokranjac et al. 2006) . As previously mentioned, the biosynthesis of chlorophyll in plants requires the light-dependent enzyme POR1 to be post-translationally imported into the plastid (Aronsson et al. 2003) . It is possible that the J-like domain found in GhCPP1-A0A1U8HKT6 may allow it to partner with an as yet unknown co-chaperone to function similarly to the TIM16/TIM14 complex, chaperoning the translocation of POR1 into the plastid. This is supported by an overlay ( **Figure 1F )** of the GhCPP1-A0A1U8HKT6 J-like domain (transparent ConSurf colored surface) with the J-like domain of TIM16 (light blue ribbon, PDB 2GUZ) that shows a highly conserved surface in GhCPP1-A0A1U8HKT6 at the modeled protein-protein interaction site with the TIM14 protein (dark blue ribbon).

Conclusion

Evidence from protein sequence, homology, and structure support that GhCPP1-A0A1U8HKT6 is likely a chaperone-like protein of POR1 in *Gossypium hirsutum* . GhCPP1-A0A1U8HKT6 is predicted to be anchored in the plastid membrane of the chloroplast where it could interact with POR and support its entry into the chloroplast where photochemical reactions of chlorophyll biosynthesis occur, similar to Arabidopsis CPP1 to which it has high sequence similarity. The molecular function of the J-like domain is still unclear. Similarity in structure to the J domain suggests a similar protein-protein interaction domain function as in the “holdase” activity of molecular chaperones, although the lack of a HPD motif indicates different binding partners. This is supported by the J-like domain in mitochondrial TIM16 being part of a heterodimer complex. It would be interesting for further studies to explore potential binding partners of GhCPP1-A0A1U8HKT6.

## Extended Data

Description: ConSurf sequence conservation results. Resource Type: Dataset. DOI: 10.22002/a9fcw-h5n51
